# Local and Systemic Responses to Low‐Intensity Cycling With Blood Flow Restriction Compared to High‐Intensity Cycling: A Randomized Crossover Study

**DOI:** 10.1111/sms.70157

**Published:** 2025-10-28

**Authors:** Sanghyeon Ji, Michael Boschmann, Michael Behringer, Patrick Wahl, Alexander Franz

**Affiliations:** ^1^ Section Exercise Physiology German Sport University Cologne Cologne Germany; ^2^ The German Research Center for Elite Sport Cologne Germany; ^3^ Experimental and Clinical Research Center Charité Universitätsmedizin Berlin and Max Delbruck Center for Molecular Medicine Berlin Germany; ^4^ Department of Sports Sciences Goethe University Frankfurt Frankfurt Germany; ^5^ Department of Orthopedics and Trauma Surgery University Hospital Bonn Bonn Germany; ^6^ Department of Trauma and Orthopedic Surgery BG Klinikum Ludwigshafen Ludwigshafen Germany; ^7^ Department of Adult Reconstruction ATOS Orthoparc Clinic Cologne Cologne Germany

**Keywords:** aerobic exercise, blood‐flow‐restricted exercise, hyperkalemia, interstitial fluid, mechanical stress, metabolic stress

## Abstract

Despite growing interest in blood flow restriction (BFR) for enhancing training adaptations, its acute impacts on local and systemic physiological stress remain incompletely understood. This study compared the metabolic and perceptual responses of low‐intensity cycling (LI) with BFR (LI + BFR) to both LI and high‐intensity (HI) cycling without BFR, matched for time and external work. Ten males (26.9 ± 4.6 years) completed LI (20 min at 55% peak aerobic power output, PPO), LI + BFR (with 50% limb occlusion pressure), and HI (10 × 1 min at 90% PPO interspersed with 1‐min recovery at 20% PPO) protocols in a randomized cross‐over design. Interstitial metabolic responses were assessed via microdialysis in the vastus lateralis; systemic blood responses were evaluated via venous blood gas analysis. Cardiorespiratory responses, including heart rate, oxygen uptake, and ventilation, were continuously monitored during exercise. Serum creatine kinase (CK) and lactate dehydrogenase (LDH) were measured as indirect markers of muscle damage, and perceptual responses were documented. Muscle interstitial lactate and pyruvate were highest in HI, followed by LI + BFR, and lowest in LI (*p* < 0.05). Systemic blood and cardiorespiratory responses were comparable between LI + BFR and HI and exceeded LI (*p* < 0.05), while electrolyte shifts occurred across all conditions (*p* < 0.001) without between‐condition differences. All protocols increased CK and LDH 24–48 h post‐exercise, with the greatest increases in HI (*p* < 0.05). Perceived exertion and pain were higher in LI + BFR than in other conditions (*p* < 0.05). In conclusion, BFR intensifies local and systemic stress during LI and may be a potent strategy to promote muscle adaptive stimulus. However, when time and total external work are matched, high mechanical loading appears more effective in inducing local stress, which may be essential for further muscular adaptation processes.

## 1 Introduction

Blood flow restriction (BFR) training has gained considerable attention for its potential to promote exercise‐induced physiological adaptations in various populations. BFR training typically combines low‐intensity exercise (e.g., 20%–40% of one repetition maximum, 1RM or < 50% of maximal oxygen uptake, V̇O_2_peak) with external occlusion via an inflatable cuff or elastic band applied proximally to the trained limb, thereby increasing physiological demands despite markedly lower mechanical loading (i.e., external loads) [1, 2]. In terms of acute responses, endurance exercise with BFR has been shown to induce pronounced muscle hypoxia accompanied by metabolic perturbations (e.g., altered acid–base balance, lactate accumulation) and oxidative stress, exceeding those of equivalent‐intensity exercise without BFR and being comparable to those induced by high‐intensity exercise [1, 3, 4, 5]. These stressors are considered potent triggers for molecular signaling pathways related to exercise‐induced angiogenesis (e.g., HIF‐1α‐VEGF signaling) and mitochondrial adaptations (e.g., AMPK‐PGC‐1α signaling) [3, 6, 7, 8, 9, 10]. Consequently, incorporating BFR into low‐ to moderate‐intensity endurance exercise has been shown to elicit physiological benefits that are comparable or even superior to conventional low‐ or high‐intensity training, including enhanced skeletal muscle oxidative capacity and homeostatic regulation [7, 11, 12, 13, 14].

Despite valuable insights from previous research into the potential of endurance exercise with BFR to enhance training stimuli, most studies have focused primarily on whole‐body (i.e., systemic) responses, such as blood parameters (e.g., pH, lactate) and cardiorespiratory variables (e.g., oxygen uptake, ventilation) [3, 4, 5, 15, 16]. In contrast, acute local responses at the muscle level during BFR exercise remain less well understood. Assessing metabolic perturbations directly at the tissue level may therefore provide meaningful clues about the mechanisms underlying BFR training‐induced adaptations, particularly as BFR creates a partially “isolated environment” in which local metabolic responses can be more distinctly observed.

The microdialysis technique is a method that has been utilized to assess metabolic dynamics within the exercising muscle. Operating on the principle of substance diffusion through a semipermeable membrane [17], this technique enables the continuous measurement of changes in e.g., metabolite concentrations within the muscle interstitial space [18, 19]. While several studies have applied microdialysis to explore exercise‐associated metabolic patterns [18, 20, 21], only one to date has investigated muscle interstitial lactate concentrations during very low‐intensity exercise (one‐leg cycling at 10–20 W) under reduced blood flow—using a lower‐body pressure chamber to apply positive pressure (30–50 mmHg) [22]. Thus, the local metabolic responses to cuff‐mediated BFR during low‐intensity exercise, particularly in comparison to conventional exercise protocols, remain insufficiently characterized.

Additionally, little is known about the potential impact of BFR on electrolyte homeostasis during exercise and exercise‐induced muscle damage. While low‐load resistance training with BFR has shown similar electrolyte responses to higher‐load exercise without BFR [23, 24], the effects of prolonged venous pooling during endurance‐type BFR exercise are insufficiently investigated. Moreover, most studies examining BFR exercise‐associated muscle damage have focused on resistance exercise, reporting mixed outcomes ranging from unchanged biomarkers to episodes of rhabdomyolysis [2, 25, 26, 27]. Understanding the effects of BFR combined with endurance exercise on muscle damage and electrolyte balance can serve as a crucial foundation for practitioners, enabling the development of targeted and safe strategies for integrating BFR into endurance training programs tailored to diverse populations.

Therefore, the present study aimed to investigate acute physiological responses to low‐intensity cycling (LI) with BFR (LI + BFR) compared to LI and high‐intensity interval cycling (HI) without BFR, matched for time and total external work. Specifically, we assessed local (muscle interstitial metabolites) and systemic responses (venous blood metabolic and ionic parameters, and cardiorespiratory variables), and indirect muscle damage markers. In addition, the subjective perception of each exercise condition was evaluated by assessing perceived exertion and muscular pain level. We hypothesized that, under conditions of matched time and total external work, low‐intensity cycling with BFR would result in elevated local and systemic physiological responses comparable to high‐intensity cycling without BFR, while inducing less muscle damage responses due to the lower mechanical loading.

## 2 Methods

### 2.1 Participants

Ten healthy young males participated in this study (Table 1). All participants engaged in regular physical activity, typically at least twice per week for ≥ 45 min per session, and were therefore classified as recreationally active [28]. Exclusion criteria included current psychological, neurological, and cardiovascular disorders that could interfere with exercise testing under the invasive study setting, as well as acute or recent musculoskeletal injuries (within the last 6 months). None of the participants had any prior experience of BFR during cycling exercise. All participants provided their informed consent by signing a document after receiving detailed verbal and written information about the study procedures and potential risks. The study was approved by the local Ethics Committee of the University Hospital Dusseldorf (Trial‐ID: 5002R) and was performed according to the Declaration of Helsinki.

**TABLE 1:** Participant characteristics and maximal physiological responses obtained during the maximal incremental cycling test.

Age (years)	26.9 ± 4.6
Height (cm)	186 ± 6.0
Body mass (kg)	79.9 ± 9.0
V̇O_2_peak (mL·min^−1^·kg^−1^)	52.8 ± 9.0
PPO (W)	376 ± 71
HR (1·min^−1^)	190 ± 6.9
V̇CO_2_ (mL·min^−1^·kg^−1^)	61.1 ± 12.7
RER	1.18 ± 0.08
V̇E (L·min^−1^)	157 ± 24.7
Blood lactate concentration (mmol·L^−1^)	11.4 ± 2.8

*Note:* Values are presented as mean ± standard deviation.

Abbreviations: HR, heart rate; PPO, peak aerobic power output; RER, respiratory exchange ratio; V̇CO_2_, carbon dioxide production; V̇E, minute ventilation; V̇O_2_peak, maximal oxygen uptake.

### 2.2 Sample Size Calculation

Based on previous studies on the physiological responses to endurance exercise with BFR [15, 29, 30], we assumed a medium to large effect size for our primary outcomes. An a priori power analysis (G*Power version 3.1.9.7) using an assumed effect size (Cohen's *f*) of 0.30, *α* = 0.05, and a statistical power (1 − *β*) of 0.90 indicated a required sample size of *N* = 6–9 for a repeated‐measures design (within–between interaction, number of measurements: 4 to 8, correlation level among repeated measures: 0.50, non‐sphericity correction: 1.0). Thus, the attained sample size of *N* = 10 can be considered adequate to detect statistically meaningful effects.

### 2.3 Experimental Design

**FIGURE 1:**
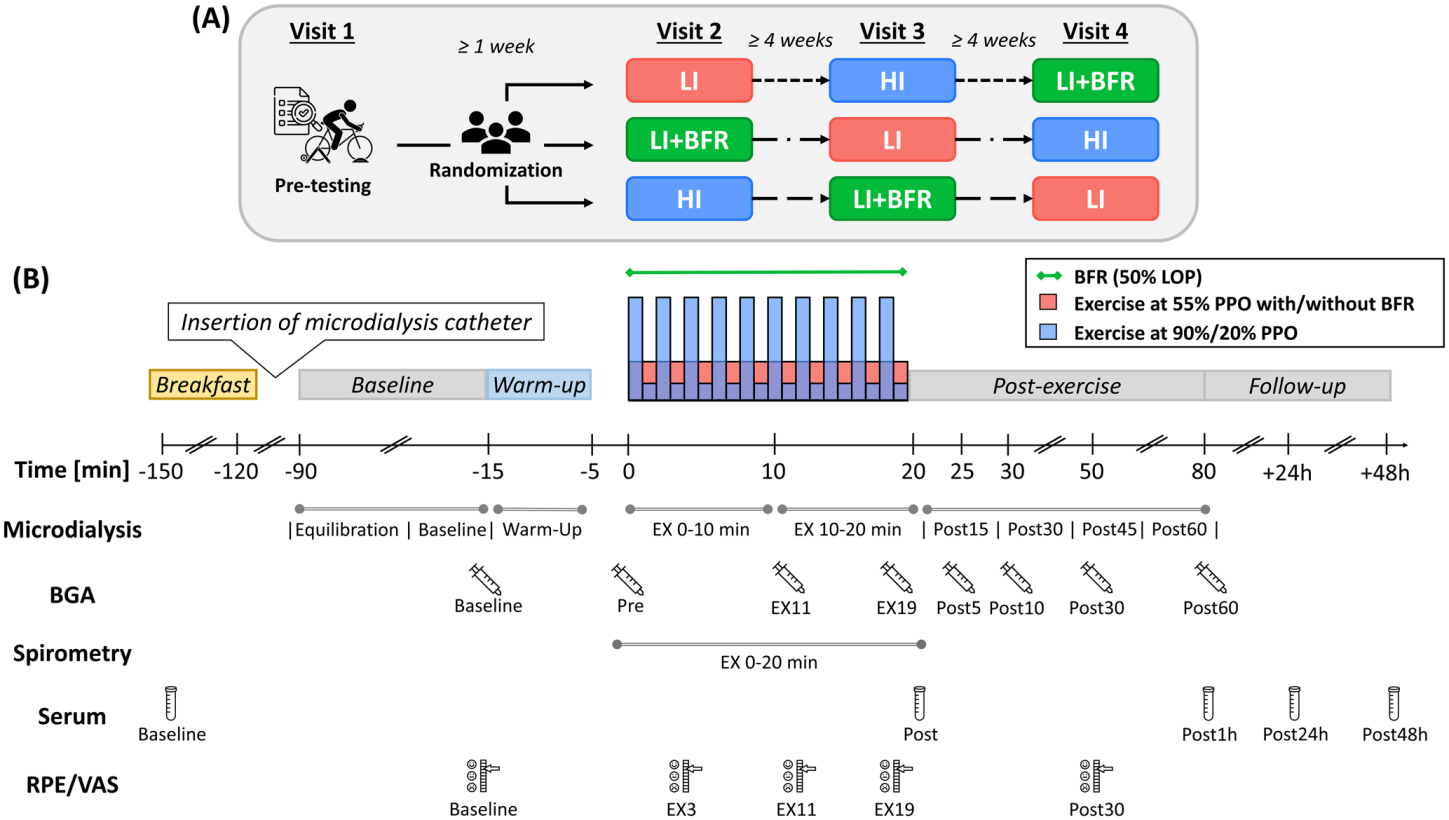
Schematic overview of the randomized within‐subject crossover study design, depicting three exercise trials in various sequences (A) and the experimental timeline, including the measurement time points (B). All participants performed three exercise trials separated by at least four weeks, consisting of low‐intensity continuous cycling (LI), LI with blood flow restriction (LI + BFR), or high‐intensity interval cycling (HI). BGA, blood gas analysis; EX, exercise; LOP, individual arterial limb occlusion pressure; PPO, individual peak aerobic power output; RPE, rating of perceived exertion; VAS, visual analog scale.

To investigate acute physiological responses to low‐intensity cycling with and without BFR compared to high‐intensity cycling without BFR matched for total work done, this study employed a randomized within‐subject crossover design. Thus, each participant completed one pre‐testing session and three experimental sessions in a randomized order, acting as their own control (Figure 1A). During the pre‐testing session, participants performed a maximal incremental cycling test followed by a familiarization with BFR cycling. Following a one‐week rest, they completed the first of three experimental sessions, each separated by at least four weeks to ensure full recovery from the previous trial and from the invasive microdialysis procedure. Participants were instructed to avoid consuming alcohol for at least 24 h and vigorous exercise for 48 h before each experiment session. All cycling tests were conducted on an electromagnetically braked ergometer (Kardiomed 520, Proxomed Medizintechnik GmbH, Alzenau, Germany).

#### 2.3.1 Pre‐Testing Session

After the measurement of body mass and height using a digital scale and stadiometer, participants completed a maximal incremental exercise test on the cycling ergometer to determine V̇O_2_peak and peak aerobic power output (PPO). The test started at a work rate of 100 W with increments of 20 W·min^−1^ until voluntary exhaustion. Throughout the test, pulmonary gas exchange and ventilatory data (MetaMax 3B, Cortex Biophysik GmbH, Leipzig, Germany) and heart rate (HR) (Polar H7, Polar Electro Oy, Kempele, Finland) were collected breath by breath and every second, respectively. Before each test, the spirometry system was calibrated with ambient air, a reference gas (5% CO_2_ and 15% O_2_), and with a 3‐L syringe, according to the manufacturer's specifications. Immediately post‐exercise, a capillary blood sample (20 mL) was taken from the earlobe to determine blood lactate concentration (Biosen C‐line; EKF Diagnostic Sales, Magdeburg, Germany).

Exhaustion was verified using the following criteria [31]: (a) HR > ± 5% of the age‐predicted maximum; (b) respiratory exchange ratio ≥ 1.10; (c) blood lactate concentration ≥ 8 mmol·L^−1^. V̇O_2_peak was defined as the highest value of a 30‐s moving average during the test. PPO was determined according to the following equation:

$$\mathrm{PPO}={\mathrm{PO}}_{\text{completed}}+t_{\text{final}}/\text{step duration}\;\text{·}\;\text{power increment}$$

where PO_completed_ is the power output at the last stage completed and *t*_final_ (s) is the time spent in the final stage.

After a 10‐min break, the individual arterial limb occlusion pressure (LOP) for each leg was determined in a supine position using an automated cuff system equipped with an internal pressure sensor (Delfi PTSII, Delfi Medical Inc., Vancouver, Canada). A 11.5 cm wide pneumatic cuff (Easi‐Fit Contour, Delfi Medical Inc., Vancouver, Canada) was positioned at the most proximal part of the thigh. The tourniquet cuff then gradually increased pressure until no further blood flow was detectable in the femoral artery, considering this pressure as the individual LOP for that leg. The same procedure was repeated for the contralateral limb. Participants then performed a 10‐min BFR‐cycling familiarization at the same intensity (55% PPO) and cuff pressure (50% LOP) as in the subsequent LI + BFR trial during the experimental sessions, in order to minimize potential confounding effects related to novelty with BFR exercise.

#### 2.3.2 Experimental Session

At each experimental session, participants arrived fasted (8–9 AM) and consumed a standardized breakfast (~60 kJ·kg^−1^; ~48% of carbohydrates, ~22% of proteins, and ~30% of fats), replicated across sessions. Water intake was also standardized (10 mL⋅kg^−1^ before, 5 mL⋅kg^−1^ after exercise).

Following breakfast, participants were positioned on a hospital bed, and a 20‐gauge venous catheter (Vasofix Safety, B. Braun, Melsungen, Germany) was inserted into the antecubital forearm vein and connected to saline‐filled tubing. Thereafter, the M 71 high cut‐off microdialysis catheter (μDialysis AB, Stockholm, Sweden) was inserted into the *Vastus lateralis* under local anesthesia (see details below). After a 75‐min baseline period, participants commenced with a 10‐min warm‐up at 40% PPO, followed by one of three different 20‐min cycling trials. A 5‐min passive rest was provided between the warm‐up and main cycling trials to allow for pre‐measurements and for cuff preparation in the LI + BFR condition. Post‐exercise measurements were conducted during a 60‐min recovery period. In addition, follow‐up measurements took place at 24 and 48 h after each experimental session. The experimental protocol and measurement time points are schematically illustrated in Figure 1.

#### 2.3.3 Cycling Trials

In each experimental session, participants completed one of the following three 20‐min cycling trials: (1) low‐intensity continuous cycling at 55% PPO (LI), (2) LI with BFR (LI + BFR), and (3) high‐intensity interval cycling (HI). The HI protocol consisted of 10 sets of 1‐min intervals at 90% PPO interspersed with 1‐min of active recovery at 20% PPO. All protocols were matched for the time and total external work (mean: 286 ± 61 kJ). Participants were instructed to maintain a cadence between 80 and 90 rpm throughout exercise.

At the beginning of the experimental session for the LI + BFR trial, the individual LOP for each leg was determined in the same manner as during the familiarization session. The BFR cuffs were inflated using a rapid cuff inflator (Delfi Medical Inc., Vancouver, Canada) 30 s before starting the LI + BFR trial, maintained throughout the exercise period, and deflated immediately afterward. The average pressure applied (50% LOP) during LI + BFR was 96 ± 9 mmHg.

#### 2.3.4 Skeletal Muscle Microdialysis

The microdialysis procedure was performed under sterile conditions. The catheter was inserted into the *Vastus lateralis* ~1/3 of the distance between the superior border of the patella and spina iliaca anterior superior, 3–5 cm lateral to that point. Local anesthesia was administered via subcutaneous injection (Xylocitin 1%, Jenapharm GmbH, Jena, Germany). Thereafter, the microdialysis catheter (M 71 High cut‐off catheter, μDialysis AB, Stockholm, Sweden), with a molecular weight cut‐off of 100 kDa, was inserted into the *Vastus lateralis* parallel to the estimated pennation angle of the muscle fibers, using a removable sheath introducer (18‐gauge needle). Subsequently, the microdialysis catheter was linked to a graduated 2.5 mL syringe filled with Ringer's solution (M Dialysis AB, Stockholm, Sweden) and perfused at 2 μL⋅min^−1^ via a high‐precision syringe pump (M 107 microdialysis pump, μDialysis AB, Stockholm, Sweden).

To establish baseline calibration of the perfusion system and allow recovery from insertion trauma, a 30‐min equilibration was followed by a 45‐min baseline sampling period. Dialysate samples were collected every 15 min during the baseline and post‐exercise period, and every 10 min during exercise, including the warm‐up and the 20‐min cycling trial (see Figure 1B). It should be noted that, similar to the previous study [22], 7% of the dialysate samples were excluded from the data analysis due to blood contamination of the dialysate and/or flow arrest. These issues are likely attributable to membrane damage, potentially caused by muscle movement. As a result, the number of microdialysis samples used in generating the data varies between participants (for details on how this was handled, see Statistical Analysis). The obtained dialysate samples were initially frozen at −20°C during the experimental sessions and subsequently transferred to −80°C after each session, where they were stored until analysis. The concentrations of interstitial glucose, lactate, and pyruvate in the dialysate were measured using a Microdialysis Analyzer (ISCUS^flex^, μDialysis AB, Stockholm, Sweden).

#### 2.3.5 Venous Blood Sampling and Analysis

Venous blood samples were drawn using a 2 mL syringe anticoagulated with 50 IU balanced lithium heparin. Sampling occurred before warm‐up, at 0, 11, and 19 min of the 20‐min cycling trial, as well as at 5, 10, 30, and 60 min during the post‐exercise period (see Figure 1B). Prior to each draw, ~2 mL of blood was initially collected with a single‐use syringe and discarded. The obtained samples were analyzed directly using an automated system (GEM premier 3500, Werfen, Bedford, USA) to assess pH, base excess (BE), bicarbonate (HCO_3_^−^), potassium (K^+^), sodium (Na^+^), calcium (Ca^2+^), as well as blood lactate and blood glucose concentrations.

#### 2.3.6 Cardiorespiratory Responses

Throughout the 20‐min cycling trial, the cardiorespiratory responses—including HR, oxygen consumption (V̇O_2_), carbon dioxide production (V̇CO_2_), respiratory exchange ratio (RER; V̇CO_2_/V̇O_2_), and minute ventilation (V̇E)—were measured using the same system as during pre‐testing. To evaluate temporal changes in the cardiorespiratory variables, the mean values from three 4‐min intervals during the exercise phase (T1: 0–4 min, T2: 8–12 min, and T3: 16–20 min) were used for the following analyses. This interval length was chosen to reduce short‐term variability and to provide a consistent framework for capturing overall cardiorespiratory demand across all cycling protocols.

#### 2.3.7 Indirect Muscle Damage Biomarkers

To assess serum creatine kinase (CK) and lactate dehydrogenase (LDH) as indirect muscle damage markers, 8.5 mL of blood was collected using a vacutainer system (Becton Dickinson, Heidelberg, Germany) at baseline (before microdialysis catheter insertion), immediately post, 1, 24, and 48 h after cycling trial (see Figure 1B). After storage at 7°C for ~30 min for deactivation of coagulation factors, the blood samples were centrifuged for 10 min at 3000 rpm and 4°C (EBA200, Hettich, Mühlheim, Germany). The serum was then stored at −80°C until subsequent analysis. Serum CK and LDH levels (U L^−1^) were analyzed using an enzymatic‐photometric method (ADVIA 1800, Siemens Healthcare, USA).

#### 2.3.8 Perceptual Responses

Participants were asked to report their rating of perceived exertion (RPE) on a 6–20 Borg scale, and their leg muscle‐specific perceived pain level on a 100 mm visual analog scale (VAS), where 0 mm represented “no pain” and 100 mm “maximal pain”. Both RPE and VAS were obtained immediately before warm‐up, at 3, 11, and 19 min during the 20‐min cycling trial, and at 30 min post‐exercise (see Figure 1B).

### 2.4 Statistical Analysis

Statistical analyses were performed using R (v4.2.2, R Foundation for Statistical Computing, Vienna, Austria). Homoscedasticity and normality were assessed through visual inspection of residual and Q‐Q plots. Changes in outcome measures over time (fixed effect with 4–8 levels) across exercise conditions (fixed effect with 3 levels: LI, LI + BFR, and HI) were analyzed using linear mixed‐effects models (*lme4 package*), which are robust to small sample sizes with slight variations and repeated‐measures designs. RPE, an ordinal variable, was analyzed using cumulative link mixed models (*ordinal package*). Each participant's baseline value was included as a random intercept (i.e., random effect factor) to account for both inter‐ and intra‐individual variability. Post hoc comparisons were conducted only when significant main effects or interactions were present, with Bonferroni correction applied (*emmeans package*). For all results, statistical significance was set at *α* = 0.05. Data are expressed as the mean ± standard deviation unless otherwise stated.

## 3 Results

### 3.1 Muscle Interstitial and Blood Metabolites

**FIGURE 2:**
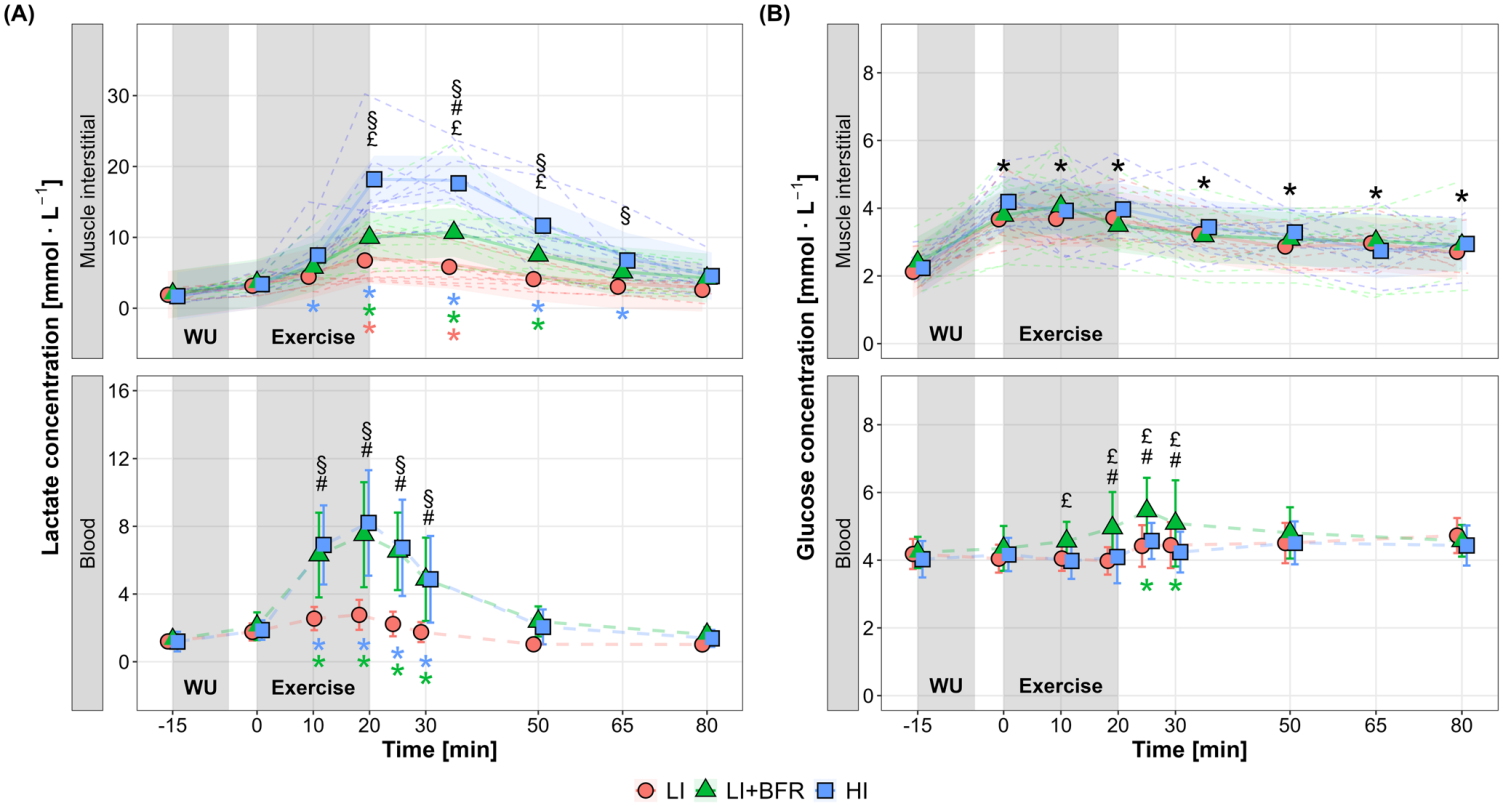
Changes in muscle interstitial and blood lactate (A) and glucose (B) concentrations in response to low‐intensity continuous cycling (LI), LI with blood flow restriction (LI + BFR), and high‐intensity interval cycling (HI), measured from baseline (−15 min) to 60 min post‐exercise (80 min). The gray shaded areas indicate the warm‐up (WU) and exercise periods. Note that the interstitial values are represented by individual data separated by condition (dashed lines) and mean (solid lines) estimated along with 95% confidence intervals (shaded areas) derived from mixed‐effects models. ^**§**^ *p* < 0.05, between LI and HI; ^**#**^ *p* < 0.05, between LI and LI + BFR; ^**£**^ *p* < 0.05, between LI + BFR and HI; *p < 0.05, difference from baseline, with colored symbols representing time effects within each condition and black symbols indicating the overall main time effect across conditions.

Muscle interstitial (Figure 2A, above) and blood lactate concentrations (Figure 2A, below) showed significant main effects for time, condition, and their interaction (*p* < 0.001). HI elicited the highest increases in interstitial lactate concentration, with significantly higher levels compared to LI + BFR (*p* ≤ 0.01) and LI (*p* ≤ 0.02) from the end of exercise to 30 and 45 min of recovery, respectively. Between LI and LI + BFR, a significant difference in muscle interstitial lactate concentration was observed only at 15 min of recovery (*p* = 0.003). Blood lactate concentration increased significantly during exercise in LI + BFR and HI and remained elevated until 10 min post‐exercise (*p* < 0.001 vs. baseline), whereas LI showed no significant change (*p* ≥ 0.34). Consequently, LI + BFR and HI exhibited significantly higher blood lactate concentrations compared to LI from 11 min into exercise to 10 min of recovery (*p* < 0.001), while no significant differences were found between LI + BFR and HI at any time point (*p* ≥ 0.79).

Muscle interstitial glucose concentration (Figure 2B, above) increased over time (*p* < 0.001), with no condition (*p* = 0.14) or interaction effects (*p* = 0.90), and remained elevated throughout the recovery period (*p* ≥ 0.03 vs. baseline). In contrast, blood glucose concentration (Figure 2B, below) showed significant time, condition, and interaction effects (*p* ≤ 0.02). Only LI + BFR resulted in increased blood glucose during exercise, with significantly higher levels from 11 min into exercise to 10 min of recovery compared to both LI (*p* ≤ 0.02, except at 11 min of the exercise period, *p* = 0.10) and HI (*p* ≤ 0.05).

**FIGURE 3:**
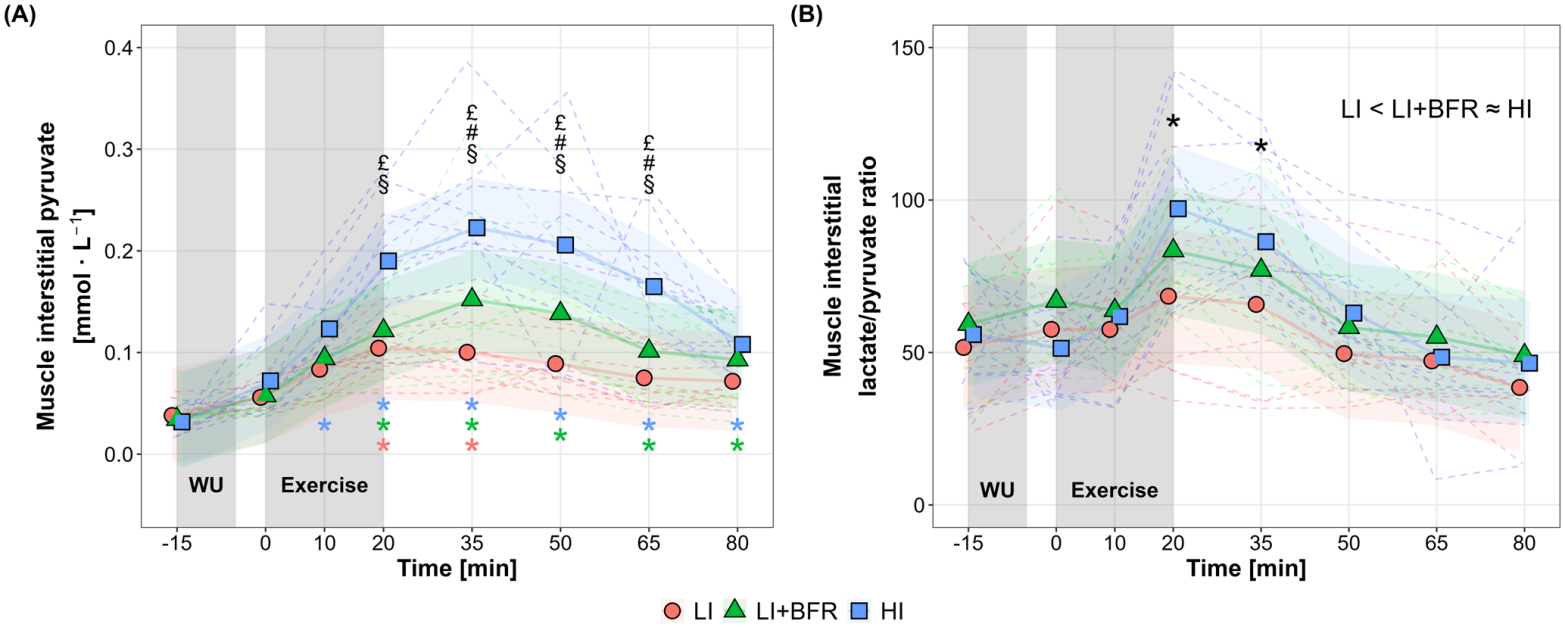
Changes in muscle interstitial pyruvate concentration (A) and lactate/pyruvate ratio (B) in response to low‐intensity continuous cycling (LI), LI with blood flow restriction (LI + BFR), and high‐intensity interval cycling (HI), measured from baseline (−15 min) to 60 min post‐exercise (80 min). The gray shaded areas indicate the warm‐up (WU) and exercise periods. Note that the values are represented by individual data separated by condition (dashed lines) and mean (solid lines) estimated along with 95% confidence intervals (shaded areas) derived from mixed‐effects models. ^**§**^ *p* < 0.05, between LI and HI; ^**#**^ *p* < 0.05, between LI and LI + BFR; ^**£**^ *p* < 0.05, between LI + BFR and HI, *p < 0.05, difference from baseline, with colored symbols representing time effects within each condition and black symbols indicating the overall main time effect across conditions. Overall main condition effect across time points is depicted above individual figures, < (*p* < 0.05) and ≈ (*p* > 0.05).

For muscle interstitial pyruvate concentration (Figure 3A), significant main effects were found for time, condition, and their interaction (*p* < 0.001). HI elicited the greatest increases, with significantly higher pyruvate levels compared to other cycling protocols from the end of exercise to 45 min of recovery (*p* ≤ 0.004). LI + BFR also resulted in significantly higher interstitial pyruvate concentrations than LI between 15 and 30 min into recovery (*p* ≤ 0.03). The interstitial lactate/pyruvate ratio (Figure 3B) showed significant time and condition effects (*p* < 0.001), but no significant interaction (*p* = 0.22). The lactate/pyruvate ratio increased significantly from the end of exercise to 15 min of recovery across all conditions (*p* < 0.001 vs. baseline), with higher values in HI and LI + BFR versus LI (*p* < 0.01).

### 3.2 Venous Blood Gas and Electrolytes

**FIGURE 4:**
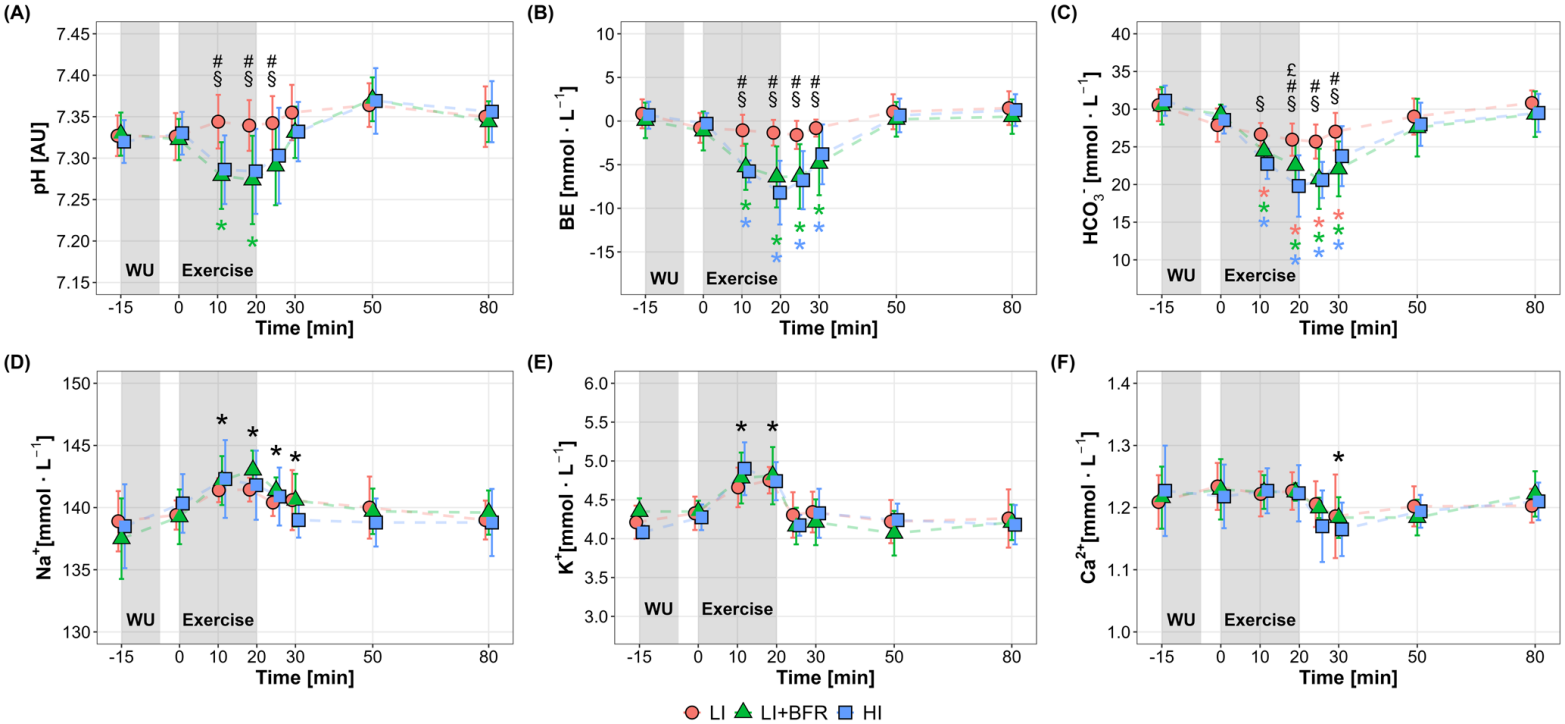
Changes in venous blood gas measures (A–C) and electrolyte concentrations (D–F) in response to low‐intensity continuous cycling (LI), LI with blood flow restriction (LI + BFR), and high‐intensity interval cycling (HI), measured from baseline (−15 min) to 60 min post‐exercise (80 min). The gray shaded areas indicate the warm‐up (WU) and exercise periods. Panels represent: (A) pH, (B) base excess (BE), (C) bicarbonate (HCO_3_^−^), (D) sodium (Na^+^), (E) potassium (K^+^), and (F) calcium (Ca^2+^). ^**§**^ *p* < 0.05, between LI and HI; ^**#**^ *p* < 0.05, between LI and LI + BFR; ^**£**^ *p* < 0.05, between LI + BFR and HI, *p < 0.05, difference from baseline, with colored symbols representing time effects within each condition and black symbols indicating the overall main time effect across conditions.

Blood gas measurements (Figure 4A–C) exhibited significant effects of time, condition, and their interactions (*p* < 0.001). Both LI + BFR and HI elicited a more pronounced decrease in blood pH, BE, and HCO_3_^−^ during exercise compared to LI, with significantly lower levels from 11 min into exercise (except for HCO_3_^−^ between LI and LI + BFR, *p* = 0.13) to 5 min (for blood pH, *p* ≤ 0.02) or 10 min of recovery (for BE and HCO_3_^−^, *p* ≤ 0.04). Between LI + BFR and HI, no significant difference (*p* ≥ 0.37) was observed except for HCO_3_^−^ at 19 min of exercise (*p* = 0.04). Following 30 min of recovery, all blood gas parameters returned to baseline levels across all conditions (*p* ≥ 0.54).

Electrolyte concentrations (Figure 4D–F) showed significant time effects (*p* < 0.001), but no condition and interaction effects (*p* ≥ 0.09). Na^+^ and K^+^ increased during exercise (*p* < 0.001 vs. baseline level), returning to baseline levels after 5 min (for K^+^, *p* = 1.00) or 30 min (for Na^+^, *p* ≥ 0.31) of recovery. Ca^2+^ remained largely unchanged across all conditions (*p* ≥ 0.19), except for a decrease at 10 min of recovery (*p* = 0.001).

### 3.3 Cardiorespiratory Responses

Table 1 summarizes key cardiorespiratory responses at baseline and during each 4‐min exercise block (see Figure S1 for additional variables). Significant main effects for time (*p* < 0.001; except for RER, *p* = 0.66), condition (*p* ≤ 0.002), and their interactions (*p* < 0.05; except for HR and V̇O_2_, *p* ≥ 0.42) were found for all cardiorespiratory variables. Overall, LI + BFR and HI resulted in comparable cardiorespiratory responses, both significantly exceeding LI (for details, see Table 1 and Figure S1).

### 3.4 Indirect Biomarkers of Muscle Damage

**FIGURE 5:**
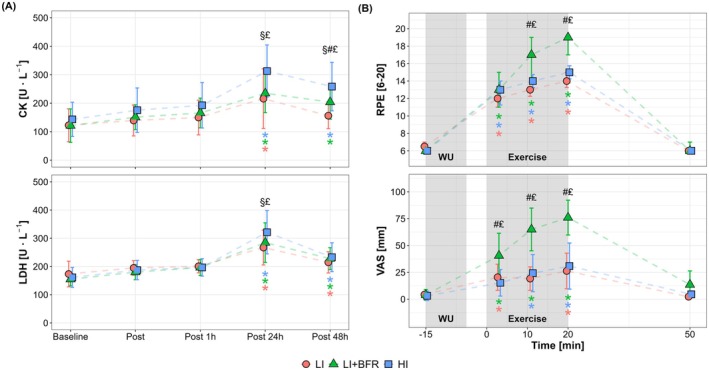
Changes in serum creatin kinase (CK) and lactate dehydrogenase (LDH) concentrations (A) and subjective perception and muscular pain, assessed by rating of perceived exertion (RPE) and visual analog scale (VAS) (B), in response to low‐intensity continuous cycling (LI), LI with blood flow restriction (LI + BFR), and high‐intensity interval cycling (HI). The gray shaded areas indicate the warm‐up (WU) and exercise periods. Note that RPE values are presented as median ± interquartile range. ^**§**^ *p* < 0.05, between LI and HI; ^**#**^ *p* < 0.05, between LI and LI + BFR; ^**£**^ *p* < 0.05, between LI + BFR and HI, *p < 0.05, difference from baseline, with colored symbols representing time effects within each condition and black symbols indicating the overall main time effect across conditions.

For indirect muscle damage markers (Figure 5A), we observed significant main effects for time and condition (except for LDH, *p* = 0.22), as well as their interactions (*p* ≤ 0.03). HI led to greater increases in CK and LDH at 24 h post‐exercise, with significantly higher levels than both LI and LI + BFR (*p* < 0.001). At 48 h post‐exercise, there were significant differences in CK among all cycling conditions (*p* ≤ 0.04), with elevated levels observed only in HI and LI + BFR compared to the baseline (*p* < 0.001). LDH remained elevated at 48 h post‐exercise with significantly higher levels compared to baseline (*p* < 0.05), with no difference between conditions (*p* > 0.57).

### 3.5 Perceptual Responses

Perceived exertion (Figure 5B, above) and muscular pain (Figure 5B, below) showed significant time, condition, and interaction effects (*p* < 0.001). LI + BFR elicited a more pronounced increase in RPE (*p* ≤ 0.03) and VAS scores (*p* < 0.001) during exercise compared to other conditions. Muscular pain levels returned to baseline by 30 min post‐exercise (*p* = 1.00), with no significant differences between conditions (*p* ≥ 0.22).

## 4 Discussion

This study aimed to characterize acute local and systemic physiological responses to low‐intensity cycling with BFR (LI + BFR) compared to low‐ and high‐intensity cycling (LI and HI), matched for time and total external work. The main findings were: (1) LI + BFR elicited significantly greater local and systemic physiological stress compared to LI; (2) although systemic responses were comparable between LI + BFR and HI, local metabolic stress induced by LI + BFR did not reach the magnitude observed during HI; (3) LI + BFR induced markedly higher perceived exertion and muscular pain compared to free‐flow conditions; and (4) all cycling protocols resulted in increased CK and LDH, with the most pronounced increases following HI.

### 4.1 Local Physiological Responses

BFR during low‐intensity cycling resulted in a more pronounced increase in local metabolic stress compared to the intensity‐matched non‐BFR cycling, as indicated by elevated muscle interstitial lactate (Figure 2A) and pyruvate concentrations (Figure 3A). This aligns with previous findings showing increased muscle interstitial lactate accumulation during unilateral leg extension under restricted blood flow (via a lower‐body pressure chamber) [22]. The increased local metabolic stress probably reflects increased reliance on anaerobic energy pathways [32, 33], driven by BFR‐induced hypoxia within the working muscle and impaired metabolite clearance [3, 5]. Similarly, Christiansen et al. [3] reported increased intramuscular lactate accumulation alongside greater muscle deoxygenation during moderate‐intensity interval running with BFR, comparable to responses under hypoxic conditions (with a FiO_2_ of 14.0%, corresponding to ~3250 m altitude).

Despite this increased local stress, muscle interstitial lactate and pyruvate accumulation during LI + BFR remained lower than HI, suggesting that our BFR exercise protocol induced less local metabolic stress than by time‐ and external work‐matched high‐intensity interval training without BFR. These findings align with Suga et al. [34], who showed greater intramuscular stress (pH reduction and phosphocreatine depletion) during low‐load resistance exercise (20% 1RM) with BFR compared to low‐load exercise without BFR, yet remained below that of high‐load exercise (65% 1RM) without BFR. Their protocols, however, were matched for total time (2 min) but not for external work, so the greater workload of high‐load exercise likely contributed to the larger intramuscular metabolic perturbations. More recently, Okita et al. [35], demonstrated that when total work volume was equalized, the intramuscular stress at the end of BFR exercise was similar across different loads (10%–40% 1RM) and exceeded that of high‐load exercise (65% 1RM) without BFR, emphasizing the dominant role of total work under BFR. In our study, both time and total external work were matched across the cycling protocols; however, HI involved intermittent bouts of higher mechanical work‐rate (i.e., J s^−1^), which likely led to greater muscle mass involvement and/or type II fiber recruitment, along with increased reliance on fast glycolytic pathways for rapid energy production, thereby enhancing intramuscular metabolite production (i.e., high metabolic turnover rate) [10, 36]. In contrast, the lower work‐rate during LI + BFR may have favored type I fiber recruitment and imposed a lower overall anaerobic load despite local hypoxia, thereby explaining why intramuscular metabolic stress was elevated compared to LI but remained lower than in HI. Collectively, our data suggest that while BFR intensifies local metabolic stress during low‐load cycling, it could not fully replicate the local metabolic environment elicited by time‐ and external work‐matched high‐intensity training. Importantly, our findings underscore that mechanical work‐rate—particularly in the context of endurance exercise—is a crucial determinant of local metabolic stress.

### 4.2 Systemic Physiological Responses

Interestingly, although local metabolic stress was lower in LI + BFR, systemic responses—including cardiorespiratory demands (e.g., increased HR, V̇O_2_, and V̇E) and blood acid–base disturbances (e.g., decreased pH, BE, HCO_3_^−^, and increased blood lactate)—were comparable to those observed during HI, both exceeding LI (Table 2 and Figure 4A–C). These findings suggest that BFR increases systemic stress without high mechanical loading, supporting previous findings on augmented cardiovascular and metabolic responses during low‐intensity aerobic exercise with BFR [3, 4, 5, 15, 29, 30]. As noted above, LI + BFR intensified local metabolic perturbations, probably caused by a combination of hypoxic environment and metabolite accumulation within the working extremity [3, 23]. This local environment may trigger chemoreceptors within the muscle (i.e., metaboreflex), potentially contributing to the increased HR and V̇E observed in LI + BFR [37].

**TABLE 2:** Cardiorespiratory responses at baseline and during every 4‐min block (T1: 0–4 min, T2: 8–12 min, and T3: 16–20 min) during low‐intensity cycling (LI), LI with blood flow restriction (LI + BFR), and high‐intensity interval cycling (HI).

	Baseline	T1	T2	T3	*p*‐Values from mixed effects models		
					Time	Condition	Time × Condition
*HR [1·min* ^*−1*^ *]*							
LI	84 ± 11	135 ± 12	148 ± 11	152 ± 10	**< 0.001** (T0 < T1 < T2 ≈ T3)	**< 0.001** (LI < LI + BFR ≈ HI)	0.423
LI + BFR	85 ± 10	135 ± 12	154 ± 15	162 ± 18			
HI	87 ± 15	146 ± 12	155 ± 11	159 ± 12			
*V̇O*_*2*_ *[mL·min* ^*−1*^·*kg* ^*−1*^ *]*							
LI	5.41 ± 1.82	26.1 ± 6.4	31.5 ± 7.3	32.6 ± 7.4	**< 0.001** (T0 < T1 < T2 ≈ T3)	**0.002** (LI < LI + BFR ≈ HI)	0.424
LI + BFR	6.09 ± 1.31	27.3 ± 5.0	34.1 ± 6.5	35.7 ± 5.8			
HI	6.29 ± 1.46	30.5 ± 5.8	33.9 ± 7.2	35.0 ± 7.6			
*V̇CO*_*2*_ *[mL·min* ^*−1*^·*kg* ^*−1*^ *]*							
LI	5.22 ± 1.72	24.2 ± 5.8^a^, ^b^	30.1 ± 7.0^a^, ^b^	31.2 ± 7.3^a^, ^b^	**< 0.001**	**< 0.001**	**0.027**
LI + BFR	6.23 ± 1.64	27.1 ± 5.2^§^, ^a^, ^b^	34.6 ± 6.8^#^, ^a^, ^b^	36.1 ± 5.5^#^, ^a^, ^b^			
HI	6.36 ± 1.87	32.0 ± 6.1^#^, ^b^	34.9 ± 7.3^#^, ^b^	35.9 ± 7.8^#^, ^b^			
*RER*							
LI	0.97 ± 0.06	0.93 ± 0.03	0.96 ± 0.03	0.96 ± 0.03	0.663	**< 0.001**	**0.028**
LI + BFR	1.02 ± 0.08	0.99 ± 0.02^§^, ^#^	1.01 ± 0.04^#^	1.02 ± 0.04^#^			
HI	1.00 ± 0.08	1.05 ± 0.03^#^	1.04 ± 0.03^#^	1.03 ± 0.03^#^			
*V̇E [L·min* ^*−1*^ *]*							
LI	17.6 ± 5.0	51.4 ± 7.5^a^	66.0 ± 10.9^a^, ^b^	68.4 ± 11.4^a^, ^b^	**< 0.001**	**< 0.001**	**< 0.001**
LI + BFR	20.3 ± 4.1	61.3 ± 9.5^a^	84.6 ± 16.9^#^, ^a^, ^b^	97.3 ± 19.2^#^, ^a^, ^b^, ^c^			
HI	20.3 ± 4.1	69.1 ± 9.6^#^, ^a^	82.8 ± 16.4^#^, ^a^, ^b^	91.5 ± 21.9^#^, ^a^, ^b^			

*Note:* Values are presented as mean ± standard deviation. Bold values indicate statistically significant results (*p* < 0.05) from the linear mixed‐effects model. Significant main effects for time and condition are denoted as < (*p* < 0.01) or ≈ (*p* > 0.05).

Abbreviations: HR, heart rate; RER, respiratory exchange ratio; V̇CO_2_, carbon dioxide production; V̇E, minute ventilation; V̇O_2_, oxygen consumption.

^#^ Significantly different versus LI (*p* < 0.05).

^§^ Significantly different versus HI (*p* < 0.01).

^a^ Significantly different versus T0 (*p* < 0.001).

^b^ Significantly different versus T1 (*p* < 0.05).

^c^ Significantly different versus T2 (*p* < 0.05).

Notably, most of the previous studies have shown generally less pronounced systemic responses during BFR exercise compared to high‐intensity exercise protocols [4, 5, 15, 29, 30], which contrasts with our findings. This discrepancy may be attributed to methodological differences. Specifically, our LI + BFR protocol was performed at a relatively higher intensity (55% PPO) compared to previous investigations (30%–40% PPO), whereas the exercise intensity for the HI protocol was similar (90% vs. 85%–95% PPO). Furthermore, previous studies often employed interval‐based exercise protocols for all experimental conditions, resulting in approximately two to three times greater total work done in HI trials compared to LI + BFR [4, 5, 15, 29, 30]. In contrast, the present investigation used a continuous cycling protocol for LI and LI + BFR and an interval‐based cycling protocol for HI, allowing matched time and total external work across conditions. These may partly explain the similar systemic physiological responses observed between LI + BFR and HI.

Importantly, our data also highlight a dissociation between local and systemic responses, underscoring that systemic markers (e.g., blood lactate) may not necessarily reflect the local metabolic state—especially in BFR or intermittent high‐intensity exercise. Supporting this, previous research has shown poor correlations between blood and intramuscular lactate concentrations during intermittent exercise [38], likely due to the continuous release and clearance of lactate into and from circulation during exercise, and differences in lactate removal rate between blood and muscle [39, 40]. Furthermore, active recovery between high‐intensity intervals has been found to reduce metabolite accumulation in the blood (e.g., H^+^ and lactate) [41]. Therefore, it is plausible to speculate that the active recovery periods with very low intensity during our HI protocol have allowed for greater metabolite disappearance from circulation and/or within the muscles, potentially suppressing metabolite accumulation in the blood and thereby diminishing systemic stress, despite pronounced local perturbations.

Taken together, our data support the “intensifying” effects of BFR on both local and systemic perturbations during low‐intensity exercise—an important stimulus for triggering molecular signaling pathways linked to skeletal muscle adaptations, such as mitochondrial biogenesis and capillarization [8, 42]. However, for a given total external work, high mechanical loading (i.e., exercise intensity) and the associated higher work‐rate appear to induce even greater local metabolic perturbations during exercise and, consequently, activate the signaling pathways essential for muscular adaptations. Nevertheless, it is also important to recognize that BFR exercise can specifically impact multiple physiological systems, either simultaneously or selectively, depending on the nature of the BFR protocol being used [1, 9]. Further research is necessary to investigate the specific impact of various endurance BFR exercise protocols on homeostatic perturbations at both the local and systemic levels, as well as their subsequent role in mediating different molecular signaling pathways that drive adaptations of different physiological systems.

### 4.3 Effects of Prolonged BFR on Electrolyte Handling and Indirect Muscle Damage Markers

With the increasing use of BFR in clinical settings, there has been growing interest in understanding its potential side effects and contraindications, particularly regarding cardiovascular, pulmonary, and muscle‐damaging responses [27, 43]. In addition to enhancing metabolic and cardiorespiratory stress, the local hypoxic environment induced by BFR exercise can also be associated with disturbed electrolyte handling, such as increased K^+^ release and/or impaired K^+^ reuptake from/into the active muscle [3, 44], which, in extreme cases, may result in an increased risk of cardiac arrhythmias [45]. However, in our study, systemic K^+^ concentration remained below the reference value (5.3 mmol⋅L^−1^) across all exercise conditions (Figure 4E). Similarly, Na^+^ and Ca^2+^ also remained within the reference ranges (Figure 4D,F), which is in line with previous findings from BFR resistance training [23, 24]. Accordingly, we can conclude that, at least in healthy individuals, there is no clinically relevant pattern of systemic electrolyte disturbances associated with prolonged venous pooling during endurance‐type BFR exercise, supporting its potential safety. Nonetheless, further research is needed to confirm whether these responses can be confirmed in clinical populations.

All exercise protocols in the present study resulted in increased serum CK and LDH concentrations, while HI induced the most pronounced responses (Figure 5A). Notably, the magnitude of muscle damage responses observed in the present study appears relatively moderate (~2–3‐fold increase after 24 h) compared to previous studies reporting ~40‐fold increases in indirect muscle damage markers following activities with more eccentric actions, such as running, eccentric cycling, and resistance exercise with and without BFR [27, 46]. Further, the responses following LI + BFR were comparable to those observed after LI, despite the markedly greater local and systemic metabolic stress. These findings suggest that the external pressure by BFR does not impose additional mechanical strain on active muscle during low‐load endurance exercise.

### 4.4 Perceptual Responses

Despite low mechanical loading, our continuous BFR protocol elicited high perceived exertion and muscular pain during exercise, even exceeding HI (Figure 5B). These observations are consistent with previous research employing continuous BFR during interval‐based exercise [5, 16, 29], suggesting that continuous occlusion may induce discomfort regardless of the exercise modality. Such perceived strain may compromise the feasibility or tolerability of continuous BFR protocols, particularly in populations with low exercise tolerance or heightened sensitivity to exertional discomfort, such as older adults, patients with chronic diseases, or individuals in early‐stage rehabilitation. In this context, cyclical BFR protocols—involving alternating periods of cuff inflation (occlusion) and deflation (reperfusion) during exercise—have emerged as an alternative with potentially greater tolerability [4, 30]. Interestingly, Corvino et al. [4] reported that cyclical BFR elicited lower perceived strain during low‐load interval exercise, while inducing higher physiological responses (e.g., blood lactate accumulation and muscle deoxygenation). However, it is important to note that the cyclical BFR protocol was performed with higher cuff pressure than the continuous BFR (~150 mmHg vs. ~100 mmHg), which makes direct comparisons difficult. Collectively, these findings emphasize the necessity of balancing physiological stimuli with tolerability, which is one of the pivotal challenges in designing BFR protocols. Given the virtually limitless combinations of BFR protocol variables (e.g., pressure, timing, and occlusion mode), it is imperative for future studies to identify optimal BFR configurations for inducing adaptations while minimizing discomfort. This will be essential for advancing BFR as a practical, individualized training strategy across a broad range of populations and settings.

### 4.5 Strengths and Limitations

This study provides novel insights into the acute physiological responses to different cycling protocols with and without BFR, using a comprehensive methodological approach that integrated invasive and non‐invasive techniques alongside subjective assessments. A key methodological strength lies in the integration of microdialysis with systemic measurements, enabling a comprehensive, multi‐level analysis of exercise‐induced stress. Furthermore, by matching all exercise conditions for both time and total external work, we sought to minimize confounding effects related to exercise volume. While this approach did not eliminate all sources of variability, it could offer a more controlled basis for interpreting the distinct physiological contributions of mechanical loading and BFR itself.

Nevertheless, some limitations should be acknowledged. Given the invasive nature of microdialysis and the participant burden associated with the complex study design, it was challenging to recruit a larger sample size. Standardizing hormonal influences related to the menstrual cycle (e.g., fluctuations in estrogen and progesterone) for female participants would have further complicated recruitment and feasibility. We therefore recruited a homogeneous cohort of young, healthy males to ensure safety and protocol adherence. While this approach enhanced internal validity, it limits the generalizability of our findings, particularly with regard to potential sex‐specific differences in physiological and perceptual responses to BFR exercise. Future studies should therefore investigate female participants and consider sex as a biological variable when evaluating acute responses to BFR. Moreover, the results cannot be directly extrapolated to other populations, such as older adults or clinical groups, which should be specifically addressed in future studies. Finally, although microdialysis enables continuous and relatively minimally invasive monitoring of the interstitial environment in close proximity to the working muscle, the resulting interstitial metabolite concentrations cannot be considered a direct proxy for intracellular environments. While this distinction must be considered when interpreting absolute values, this technique remains a valuable method for capturing muscle‐specific metabolic dynamics in response to exercise that are not detectable in systemic circulation [21].

## 5 Conclusions

BFR prominently increased physiological stress at both local and systemic levels during low‐intensity cycling. However, local metabolic disturbances induced by low‐intensity cycling with BFR were still lower than during the high‐intensity interval cycling without BFR, even under matched total external work. These findings suggest that BFR is an effective strategy to augment training stimuli of low‐intensity exercise but may not fully replicate the local metabolic stress of high‐intensity exercise. Nevertheless, our findings highlight the potential of BFR to promote muscle remodeling adaptations under reduced mechanical loading without signs of excessive muscular disruption or electrolyte disturbances. However, the high perceptual load associated with the continuous BFR protocol may compromise its feasibility or tolerability, particularly in populations with low exercise tolerance. Future work should focus on optimizing BFR protocols to balance physiological effectiveness with tolerability, thereby facilitating broader application across athletic and clinical populations.

## 6 Perspective

Our data contribute to the growing body of research supporting BFR as a strategy to enhance both local and systemic training stimuli during low‐intensity exercise. Notably, these responses occurred without inducing excessive muscle damage or electrolyte disturbances, supporting the use of BFR exercise in contexts where high mechanical loading is contraindicated, such as in clinical rehabilitation or during athletes' de‐loading and recovery phases. However, the high level of discomfort or pain associated with continuous BFR protocols highlights the need to refine BFR application strategies to optimize tolerability without compromising effectiveness. Thus, future research should explore alternative BFR configurations (e.g., intermittent occlusion pattern) to better balance between efficacy and comfort.

## Conflicts of Interest

The authors declare no conflicts of interest.

## Supporting information


**Figure S1:** sms70157‐sup‐0001‐FigureS1.docx.

## Data Availability

The data that support the findings of this study are available from the corresponding author upon reasonable request. Source data underlying all Figures and Tables are provided as a Source.
