# Supplementary material for: Local and Systemic Responses to Low‐Intensity Cycling With Blood Flow Restriction Compared to High‐Intensity Cycling: A Randomized Crossover Study
Source: Scand J Med Sci Sports. 2025 Oct 28;35(11):e70157. doi: 10.1111/sms.70157 (PMC12560157; doi:10.1111/sms.70157)
Supplement: Supplementary file 1 — Figure S1: sms70157‐sup‐0001‐FigureS1.docx. [file SMS-35-e70157-s001.docx]

# Supplementary material

**
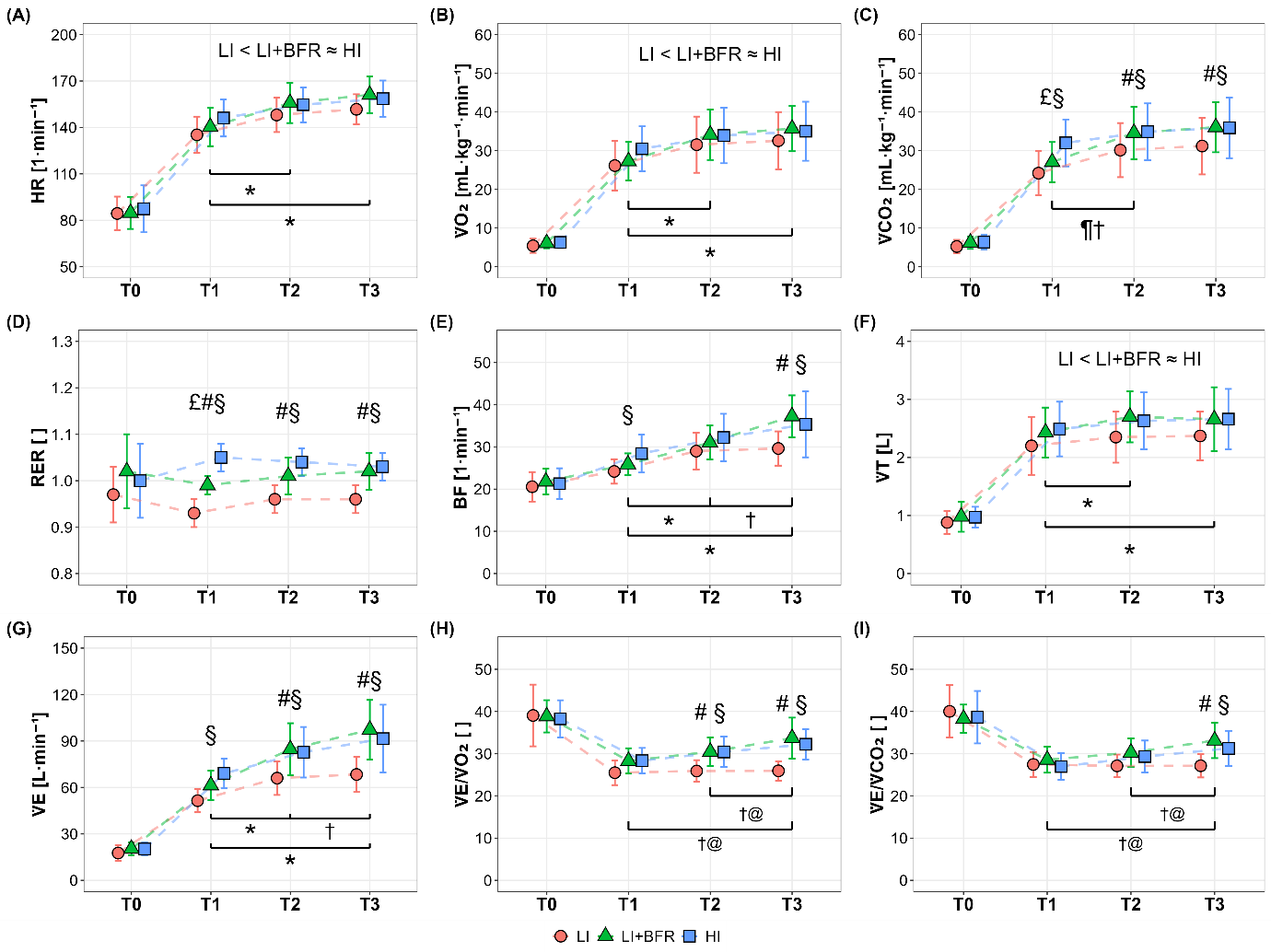
**

**Supplementary figure S1.** Cardiorespiratory responses during low-intensity continuous cycling (LI), LI with blood flow restriction (LI+BFR), and high-intensity interval cycling (HI). (A) heart rate (HR), (B) oxygen consumption (V̇O_2_), (C) carbon dioxide release (V̇CO_2_), (D) respiratory exchange ratio (RER), (E) breath frequency (BF), (F) tidal volume (VT), (G) minute ventilation (V̇E), (H) ventilatory equivalent for oxygen (V̇E/V̇O_2_), (I) ventilatory equivalent for carbon dioxide (V̇E/V̇CO_2_). Data are depicted as means ± standard deviations for the baseline (T0) and three 4-min blocks during the exercise period (T1: 0 – 4 min, T2: 8 – 12 min, and T3: 16 – 20 min). ^§^p < 0.05, between LI and HI; ^#^p < 0.05, between LI and LI+BFR; ^£^p < 0.05, between LI+BFR and HI; **^†^**p < 0.05, difference between time points within LI+BFR; ^@^p < 0.05, difference between time points within HI; Main condition effect across time points is depicted above individual figures, < (p < 0.05) and ≈ (p > 0.05); *p < 0.05, difference between time points (main time effect across conditions).
